# Controllable Photodynamic Therapy Implemented by Regulating Singlet Oxygen Efficiency

**DOI:** 10.1002/advs.201700113

**Published:** 2017-06-23

**Authors:** Wenting Wu, Xiaodong Shao, Jianzhang Zhao, Mingbo Wu

**Affiliations:** ^1^ State Key Laboratory of Heavy Oil Processing China University of Petroleum Qingdao 266580 China; ^2^ State Key Laboratory of Fine Chemicals School of Chemical Engineering Dalian University of Technology Dalian 116024 P. R. China

**Keywords:** cancer, photodynamic therapy, photosensitization, singlet oxygen

## Abstract

With singlet oxygen (^1^O_2_) as the active agent, photodynamic therapy (PDT) is a promising technique for the treatment of various tumors and cancers. But it is hampered by the poor selectivity of most traditional photosensitizers (PS). In this review, we present a summary of controllable PDT implemented by regulating singlet oxygen efficiency. Herein, various controllable PDT strategies based on different initiating conditions (such as pH, light, H_2_O_2_ and so on) have been summarized and introduced. More importantly, the action mechanisms of controllable PDT strategies, such as photoinduced electron transfer (PET), fluorescence resonance energy transfer (FRET), intramolecular charge transfer (ICT) and some physical/chemical means (e.g. captivity and release), are described as a key point in the article. This review provide a general overview of designing novel PS or strategies for effective and controllable PDT.

## 1 Introduction

**Figure 1:**
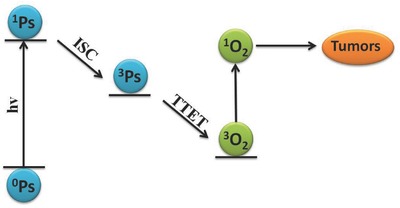
The generation of ^1^O_2_ by intersystem crossing (ISC) and triplet‐triplet energy transfer (TTET).

Photodynamic therapy (PDT) is a promising noninvasive approach for the treatment of cancer tumors by combining photosensitizer (PS), oxygen molecule and light.^1^ In clinical applications, a low toxic or non‐toxic PS is delivered to the tumor tissue and cancer cells by active or passive diffusion. Subsequently, PS is excited from its low energy ground state (S_0_) to an higher energy excited state (S_1_) by irradiating tumor tissue with long wavelength light (650–900 nm, light in this wavelength range gives good tissue penetration). In the treatment process, reactive oxygen species are responsible for destroying cancer cells, and the first singlet excited state of molecular oxygen (singlet oxygen, ^1^O_2_) is the key cytotoxic agent of PDT.^2^ ^1^O_2_ is effective in disrupting biological tissues as a result of its high reactivity.^3^ It is generated by the triplet‐triplet energy transfer (TTET) between ground‐state oxygen (triplet state) and PS (T_1_) formed by intersystem crossing (ISC, **Figure** **1**).^4^

ISC is an important non‐radiation process in the photochemical and photophysical fields, which is also a pivotal mechanism for the generation of ^1^O_2_.^5^ Usually, ISC takes place between different energy levels such as S_1_ to T_n_ or T_1_ to S_0_, and the quantum yield of ISC can be influenced by various factors such as heavy atom effect,^6^ electron configuration,^7^ perturbation of oxygen,^8^ and so on. To the best of our knowledge, the generation of ^1^O_2_ is mainly achieved by TTET, which takes from PS's triplet to ground oxygen (triplet state). Therefore it is possible to modulate the generation of ^1^O_2_ by turning ISC efficiency with disparate environments.^9^

There is no doubt that PS is essential for valid PDT.[[qv: 9b,10]] Hence, research was focused on improving the properties of PS by designing novel sensitizers. The following requirements are essential for an ideal PS: (1) low cytotoxicity and high biocompatibility,[[qv: 10a]] (2) long‐wavelength absorption,^11^ (3) selective uptake into the cancer tissues and specific release ^1^O_2_,^12^ (4) effective ISC and a long‐lived triplet excited state (high ^1^O_2_ quantum yield).^13^ However, most of traditional PS, with no selectivity and damage of normal cells, hardly meet the aforementioned criteria. Therefore, it is essential to design a novel PS which can be controlled as required, giving rise to ^1^O_2_ on/off responses in cancer and normal cell, respectively.

Recently, controllable photosensitization process, in which ^1^O_2_ are supposed to be modulated on demand, has received increasing attention.^14^ Comparing with traditional PS, only in tumor region can ^1^O_2_ be released and little normal cells are damaged. The formation of ^1^O_2_ is prerequisite for an efficiently controllable photosensitization. As mentioned before, the generation of ^1^O_2_ is directly related to ISC. Hence, the critical issue is how to regulate ISC efficiency. In fact, ISC is a kind of inactive pathway in the process of excited‐state molecular deactivation, and there are many other mechanisms promoting the inactivation of excited‐state molecules such as photoinduced electron transfer (PET),^15^ fluorescence resonance energy transfer (FRET),^16^ and intramolecular charge transfer (ICT).^17^ Two or more inactive pathways are always competed with each other during the exciton deactivation. If ISC plays a dominant role, ^1^O_2_ can be produced, that is ^1^O_2_‐ON state. Otherwise, production of ^1^O_2_ is quenched or inhibited if other pathways suppress ISC, this is ^1^O_2_‐off state. In summary, it is a novel strategy to regulate ^1^O_2_ quantum yield indirectly by modulating ISC efficiency. Of course, the physical captivity and controlled release of PS, such as delivery carriers based on the peptide and protein,^18^ are also good ways to directly control PDT process.

**Figure 2:**
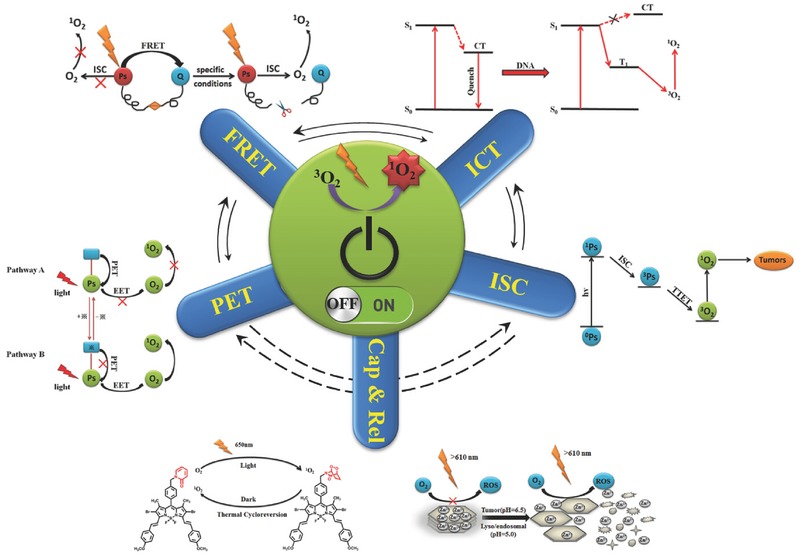
An illustration of all control mechanisms described in this review. Cap and Rel indicate factitious captivity and release mentioned in section 6.1.

Attention has been paid to efficient and selective control in the ^1^O_2_ quantum yield. In this review, we focus on the modulation of production of ^1^O_2_ based on various pathways developed since the year 2000. The categorizations of the articles are based on different mechanism of action. To better understand all control mechanisms mentioned in this work, a simple illustration and a summative comparisons table were given in **Figure** **2** and **Table** **1**. We hope to provide a general overview of designing novel PS or strategies for effective and controllable PDT.

**Table 1:** Summative comparisons of all control mechanisms mentioned in this work

Item	Control Requirements	Converted Means/Methods	Advantages	Disadvantages
Object				
PET	electron donor/acceptor	pH (acid), solvent polarity	highly effective, strong expansibility, programmable	need to specially design electron donor/acceptor, less conversion means
FRET (inhibitive/reversible)	energy donor/acceptor, FRET‐linker, spectral overlap, limited distance, energy level matching	pH (acid), enzyme, DNA, H_2_O_2_, light	versatile, readily, highly effective, programmable, strong expansibility	need to specially design energy donor/acceptor, complicated systems
ICT	electron donor/acceptor	pH (acid), solvent polarity	highly effective, programmable	need to specially design electron donor/acceptor less research
Factitious captivity and release of PS	governable and decomposable Frameworks	pH (acid)	simple mechanism, highly effective, strong expansibility, multifunction	multicomponent system, complex strategies, less conversion means
Chemical release and capture of ^1^O_2_	activatable endoperoxides	temperature	intermittent PDT, bright future	no O_2_ self‐sufficient, less research

## 2 PET Regulation Mechanism

PET^19^ can be used to modulated the generation of ^1^O_2_ when compete with others mechanisms, especially ISC. It is well known that ^1^O_2_ quantum yield is directly related to the efficiency of ISC (generally, high ISC efficiency results in high ^1^O_2_ quantum yield). Usually, PET and ISC are competitive with each other in the process of deactivation of excited states. Therefore, given charge recombination won't lead to formation of triplet state, inhibition of PET can significantly improve the efficiency of ISC, resulting in an enhancement of ^1^O_2_ quantum yield. Therefore, the generation of ^1^O_2_ can be controlled by modulating PET process.

For the controllable PET system,[[qv: 15b,20]] it is always composed of electron donor (D) and acceptor (A), which can be separated from each other in solution.^21^ Combining previous excellent works, it was shown that a PET process can be regulated by many stimulus, such as ions, pH, carbohydrates, phosphates, etc.^22^

**Figure 3:**
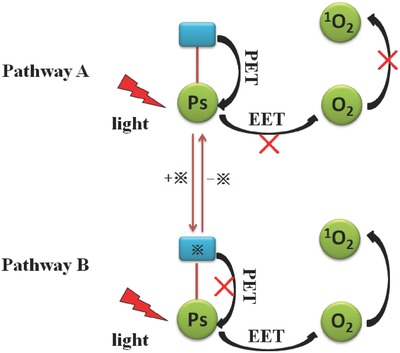
The modulation mechanism of ^1^O_2_ based on PET. Substrate‐specific receptor is shown in blue; PS is shown in green. Black cross (※) represents the substrate. Adapted with permission.^23^ Copyright 2005, American Chemical Society.

O'shea et al.^23^ reported a strategy to modulate the generation of ^1^O_2_ by PET mechanism with pH variation of the solution. ^1^O_2_ quantum yield is controlled by switching on/off of the PET process. In order to achieve such a tuning effect, amine (as a specific receptor for H^+^ and PET donor) was attached to PDT agents by covalent bond (**Figure** **3**). In the absence of the substrate (that is H^+^), the unbound receptor is with a rapid quench of the PS excited state by PET process, thereby ^1^O_2_ production is shut down (Figure 3, pathway A). In contrast, ^1^O_2_ generation would occur if acid was added, as the PET pathway would be switched off by the protonation of the amine (Figure 3, pathway B).

**Figure 4:**
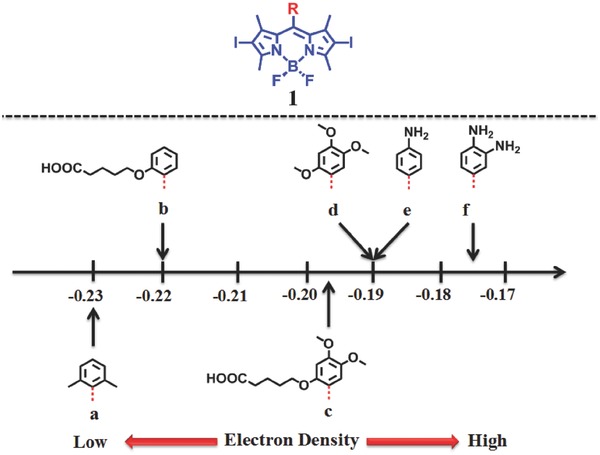
Chemical structures of 2I‐BDP derivatives and electron density of various substituent. Reproduced with permission.^24^ Copyright 2008, National Academy of Sciences.

Tetsuo Nagano et al.^24^ reported a novel strategy for controlling the generation of ^1^O_2_. The environment‐sensitive PSs (ESPers) could be active in hydrophobic (low‐polarity) environment for high ^1^O_2_ generation while little ^1^O_2_ production was observed in polar cytosolic environment (**Figure** **4**).^24^ The ESPers are supposed to bind inositol 1,4,5‐trisphosphate receptor which bears a strong‐affinity hydrophobic moiety.^25^, ^26^ In decay of the excited PS, photosensitization and PET always compete with each other, resulting in the decrease of fluorescence or ^1^O_2_. The PET process is known to depend on the highest occupied molecular orbital (HOMO) energy level of the electron donor and the solvent polarity, so they designed and synthesized a series of PS derivatives attached with various electron donor moieties.

As shown in Figure 4, **1a‐1f** were all 2I‐BDP derivatives with different substituents (methyl, methoxy and amino) as PET donor for obtaining various HOMO energy level. The ^1^O_2_ production of the compounds with high HOMO energy level can be effectively shut down by polar solvents. So the ^1^O_2_ quantum yield of these 2I‐BDP derivatives could indeed be modulated by PET. **1c–1e** could hardly generate ^1^O_2_ in solvents more polar than acetone (dielectric constant, DC ≈ 20.7), while the photosensitization ability was restored in solvents less polar than CH_2_Cl_2_ (DC ≈ 9.14). It turned out that 2I‐BDP derivatives with HOMO energy around −0.17 to −0.19 hartree could be taken as ESPers, which would be activated in a hydrophobic environment while little ^1^O_2_ was generated in a polar environment.

**Figure 5:**
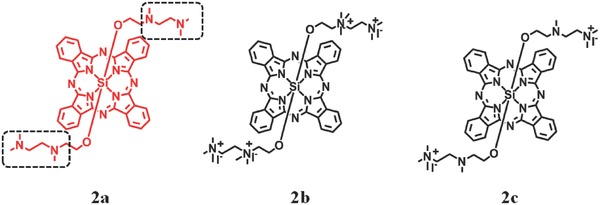
The structure of silicon‐phthalocyanines PSs. Reproduced with permission.[[qv: 22c]] Copyright 2010, The Royal Society of Chemistry.

Novel strategies which can modulate fluorescence emission and quantum yield of ^1^O_2_ by pH‐sensitive compounds have received considerable attentions.[[qv: 22c]] Phthalocyanine is an effective PS which can reveal significant acid‐dependent ^1^O_2_ generation by appropriate substitution.^27^ Ng et al. proposed a pH‐dependent ^1^O_2_ photosensitization with silicon(IV) phthalocyanine as PS (**Figure** **5**).[[qv: 22c]] Compound **2a** (B band at 352 nm, Q band at 684 nm in water) was prepared by mixing the silicon phthalocyanine dichloride with ethanol in the presence of pyridine in toluene. Compound **2b** (di‐*N*‐methylated derivative) and **2c** (tetra‐*N*‐methylated derivative) are considered as reference. The productivity of ^1^O_2_ is enhanced by 3 times when the pH reduces from 7.4 to 6.0, as a result of protonation of the amino groups which can inhibit the PET process effectively.

**Figure 6:**
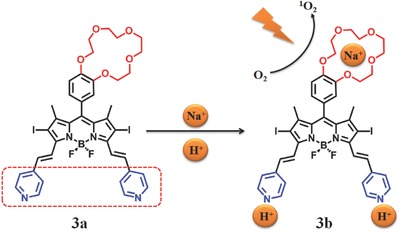
Chemical structure of BODIPY derivatives and the AND logical gate for PDT. Adapted with permission.^28^ Copyright 2009, American Chemical Society.

Another way to modulate the quantum yield of ^1^O_2_ by PET mechanism is to prepare an appropriate crown ether and pyridine substituted boron‐dipyrromethene (BODIPY, **Figure** **6**). Akkaya and co‐workers^28^ showed this BODIPY derivative could generate more ^1^O_2_ when two different signals were present under the same conditions, that is a AND logical gate.^29^ It is a remarkable fact that the pH of tumor tissues can be very low,^30^ while intracellular sodium ion concentration is almost 3 times higher than in healthy ones.^31^ So they combined crown ether with pyridyl–styryl substituents in BODIPY (**3a**) to ensure higher sensitivity for both proton and sodium ion concentrations in tumor cells.

^1^O_2_ quantum yield are enhanced by 5 times when both Na^+^ and H^+^ are present. While an apparent low rate of output was observed when neither trifluoroacetic acid (TFA) nor Na^+^ was present or only TFA was present. Herein, amino is the donor of PET and crown ether is acceptor. One interpretation for this phenomenon may be that PET channel is remarkably inhibited due to the presence of Na^+^ and H^+^, resulting in high efficiency ISC and restored ^1^O_2_ generation. In contrast, there is still a strong PET process when only TFA or Na^+^ is present. However, the experimental conditions (concentration and acid) is beyond the limits of biocompatibility normal standard. No further biological related researches were reported.

**Figure 7:**
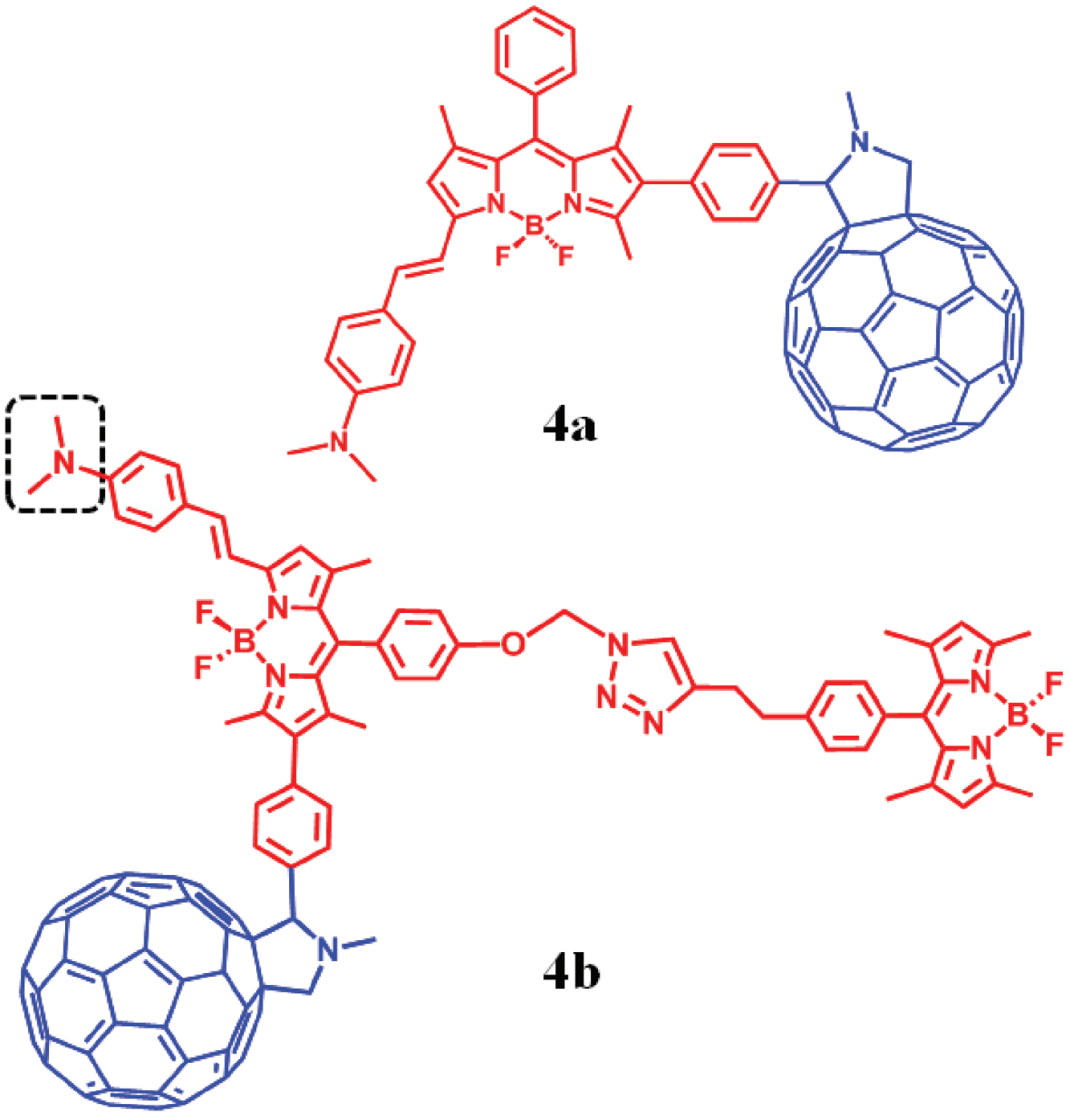
The structure of BODIPY‐C_60_ dyads (**4a**) and triads (**4b**).

By synthesizing dimethylaminostyryl BODIPY‐C_60_ dyads and triads, Zhao et al. achieved pH‐controllable modulation of ^1^O_2_ based on PET.^32^ The visible light‐trapping antenna BODIPY moieties are regarded as PET‐donor and singlet energy donor, while C_60_ groups are considered as PET‐acceptor and singlet energy acceptor (**Figure** **7**).^32^ 4a is dyads (λ_abs_ = 573 nm, λ_em_ = 591 nm) while **4b** is triads (λ_abs_ = 504/574 nm, λ_em_ = 587 nm). In the absence of acid, the S_1_ state energy level of the dimethylaminostyryl‐BODIPY moieties is higher than that of C_60_, so there is an efficient energy transfer from BODIPY to C_60_ (thus the S_1_ state of C_60_ is populated). However, the triplet excited state (T_1_) would be quenched by efficient PET from BODIPY part to C_60_. As a result, little ^1^O_2_ is generated by TTET, and this is the ^1^O_2_‐off state. In the presence of acid, PET effect will be inhibited due to the protonation of dimethylamino, so the triplet excited state of BODIPY part cannot be quenched and ^1^O_2_ would be produced by TTET, this is the ^1^O_2_‐ON state. For **4a**, the singlet oxygen quantum yield is 1.9% at neutral condition and 73% in acid. For **4b**, that is 2.0%/1.8% in neutral and 52%/63% in acid, respectively.

## 3 FRET Regulation Mechanism

**Figure 8:**
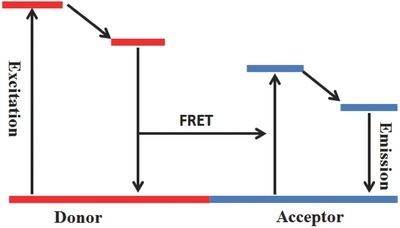
The mechanism of the FRET process.

FRET was used for the structural analysis of DNA,^33^ protein,^34^ targeted therapy,^35^ fluorescent probe,^36^ biosensors^37^ etc.^38^ When there is a spectral overlap between energy donor (D, emission spectrum) and energy acceptor (A, acceptor absorption), the fluorescence of the donor will be quenched, and the fluorescence of the energy acceptor will be observed (**Figure** **8**).^39^ FRET is achieved upon donor‐acceptor energy transfer based on a long‐range dipole–dipole interaction. Energy transfer includes two main mechanisms: (1) resonance energy transfer (RET) between the singlet state of donor and acceptor, namely Förster resonance energy transfer (FRET); (2) the other is RET between triplet state of the donor and singlet state of acceptor, that is Dexter electron transfer mechanism.^40^ FRET occurs when: (1) there is a spectral overlap between D and A; (2) proper distance between D‐A, normally less than 10 nm; (3) a relatively high quantum yield in donor.^41^ The FRET fluorescence quenching always correlates with ^1^O_2_ quenching, providing a convenient method to assess activatable photosensitizers.^42^

### 3.1 Inhibitive FRET

FRET is a kind of RET mechanism.^43^ FRET can be used to regulate the generation of ^1^O_2_ given it can compete with other excited state relaxation processes such as ISC, PET, etc. Therefore, in order to regulate ^1^O_2_ quantum yield, it is necessary to promote or decrease FRET intensity, to drain the singlet excited state of the energy donor (which is responsible for ISC and thus production of the singlet oxygen). As mentioned before, FRET can be influenced by the distance between the donor and acceptor.^44^ In order to control FRET, donor and acceptor are often combined via a special linker which can be sensitive to various specific triggers, such as some special enzymes related to tumors. Energy transfer between D and A can be terminated once the linker is cleaved by reacting with specific substrates.

**Figure 9:**
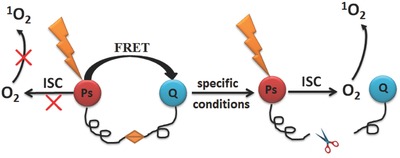
The modulation mechanism of ^1^O_2_ based on FRET. Red ball, PS; Blue ball, quencher.

Based on these mechanisms, some novel compounds combining with PSs, cleavable linker and ^1^O_2_ quencher were designed as photodynamic molecular beacons (PMB).^45^ Herein, PS is the donor of the FRET, and the quencher is the energy acceptor (**Figure** **9**). In this case, an efficient energy transfer between PS and acceptor will inhibit the singlet oxygen production. Little or no ^1^O_2_ will be generated unless the linker is cleaved by some specific conditions in tumors, namely, photosensitization is in off state in normal cells. In tumor cells, the linker will be cleaved in the presence of the specific conditions (pH value and specific enzymes); therefore PS and the quencher are separated with each other and energy transfer (FRET) will be forbidden, resulting in the restoration of the photosensitization, and ^1^O_2_ can be generated via irradiation.

**Figure 10:**
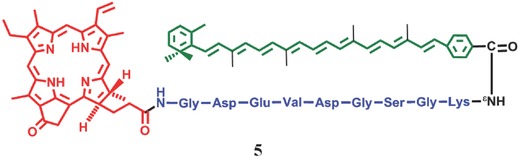
The structure of caspase‐controllable PMB. Red: pyropheophorbide (PS); Green: carotenoid (Q); Blue: caspase‐cleavable linker. Adapted with permission.^46^ Copyright 2009, American Chemical Society.

An controllable PMB **5** with caspase‐cleavable peptide as the linker was prepared by Zheng et al.,^46^ in which chlorophyll analog pyropheophorbide (absorption at 667 nm, ^1^O_2_ quantum yield >50%)^47^ was used as the PS moiety (D) while carotenoid (A) was the quencher of ^1^O_2_ and triplet excited states energy acceptor (**Figure** **10**).^46^ As mentioned above, when conveyed into target‐area, the PMB was cleaved in the presence of caspase, and the carotenoid and pyropheophorbide is no longer linked by a covalent bond, thus results in the production of ^1^O_2_; in contrast, little ^1^O_2_ can be produced in normal cells due to an effective energy transfer between pyropheophorbide and carotenoid.

**Figure 11:**
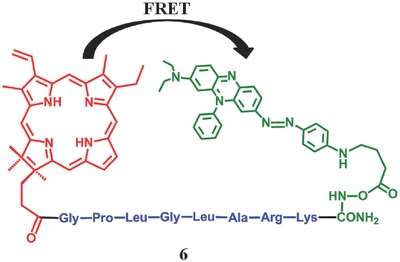
The structure of metalloproteinases‐cleavable PMB. Red: pyropheophorbide (PS); Blue: peptide sequence (linker); Green: black hole quencher‐3 (BHQ3, Q). Adapted with permission.^48^ Copyright 2007, National Academy of Sciences.

**Figure 12:**
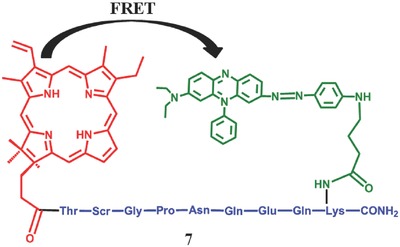
The structure of fibroblast‐cleavable PMB. Adapted with permission.^49^ Copyright 2009, American Chemical Society.

In previous studies, caspase was regard as a molecular scissor to cleave the specific peptide sequence, results in inhibition of FRET. Nevertheless, it is hard to evaluate the PDT in apoptotic cells with this agent, because caspase itself is a cell apoptosis marker. Hence, it is essential to find new enzyme and specific cleavable peptide linker for effective FRET modulation. Accordingly Zheng et al.^48^ prepared a metalloproteinases‐cleavable PMB **6**. Matrix metalloproteinases are a family of extracellular proteinases and closely related to cancer and tumor progression. Notably, a short peptide sequence, GPLGLARK, was employed as a specificity‐linker whose break can be induced by italics (**Figure** **11**). It turned out that the MMP7‐specific‐peptide linker can be cleaved in MMP7‐positive cells and the PS can be activated, resulting in 18‐fold increase in ^1^O_2_ quantum yield. Similarly, another PMB **7** with fibroblast‐cleavable linkers was synthesized by Zheng et al. in 2009 (**Figure** **12**).^49^ Herein, fibroblast activation protein is an initiator highly expressed in cancer‐related fibroblasts of human epithelial carcinomas but not in normal fibroblasts or tissues.^50^ Experiments show that peptide sequence linker could be cleaved effectively by fibroblast activation protein.

**Figure 13:**
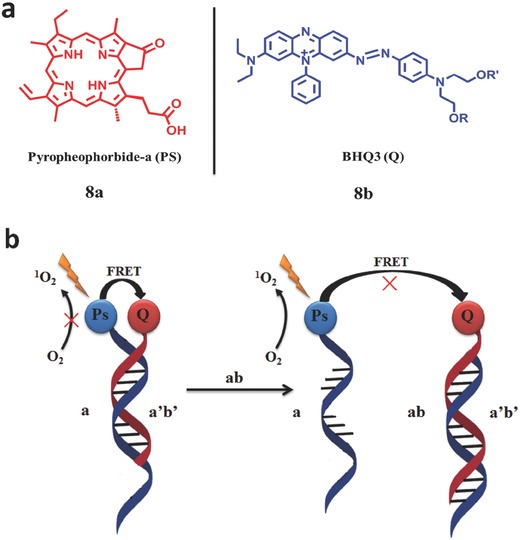
a) Structure of PS and quencher. b) the controlling mechanism of ^1^O_2_ by DNA –controllable agent. Adapted with permission.^51^ Copyright 2006, American Chemical Society.

Gothelf et al.^51^ reported a DNA sequence‐specific PDT reagent based on reverse hybridization (**Figure** **13**). Pyropheophorbide (PS, **8a**, absorption at 415 nm and fluorescence at 670 nm, ^1^O_2_ quantum yield = 0.53 ± 0.04) attached to a short 15‐mer nucleotide sequence is the PS and BHQ3 (Q, **8b**) attached to a 21‐mer oligonucleotide is the quencher (Figure 13a).^51^ By DNA‐programmed assembly, PS and Q are in proximity,^52^ resulting in an effective FRET effect which can restrain the ^1^O_2_ generation. When another DNA sequence is introduced into this system, P‐DNA linker could be replaced and released, resulting in a recovery of photosensitization, then ^1^O_2_ can be produced with irradiation (Figure 13b).^51^

**Figure 14:**
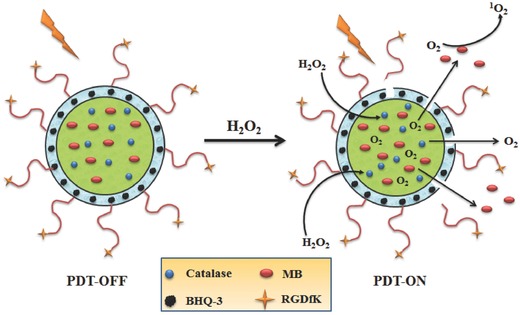
The structure of H_2_O_2_‐activatable multifunction nanoparticles and its working principles. Adapted with permission.^53^ Copyright 2015, American Chemical Society.

Guo et al.^53^ report a novel multifunction nanoparticles which can achieve targeted transport, controllable PDT and effective supply of O_2_, simultaneously. Herein, poly(d,l‐lactic‐co‐glycolic acid) (PLGA) was used as the framework of the multifunction nanoparticles; methylene blue (MB) (PS) and catalase were placed in the aqueous core, and BHQ3 doped into the PLGA shell was chosen as an quencher of PDT effect. In addition, cyclic pentapeptide incorporated on the surface of nanoparticle was regarded as the targeting ligand. (**Figure** **14**).^53^ There is an efficient FRET between MB and catalase which can restrain the in vitro generation of ^1^O_2_. The nanoparticles could be absorbed in vivo by tumors selectively. Subsequently, H_2_O_2_ enters into the core of nanoparticles, O_2_ would be produced by the catalytic action of catalase, resulting in the crack of PLGA and release of MB. Photosensitization is recovered and ^1^O_2_ is generated with the termination of FRET. Notably, the strategy could give a targeted and efficient PDT with controllable PDT and autarkic O_2_.

**Figure 15:**
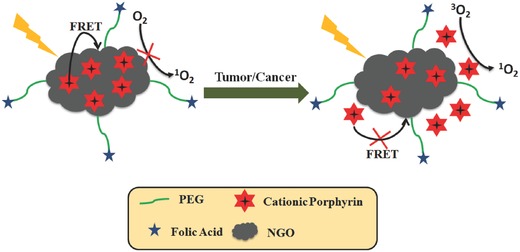
The structural representation of dual‐targeting (cellular targeting and subcellular targeting) nanosystem and its regulation mechanism. Adapted with permission.^57^ Copyright 2015, American Chemical Society.

Compared with the normal physiological pH, the tumor or cancer cell are with weakly acidic feature. This acidic environment could also be used to control the FRET process in PDT. It has been demonstrated that nanographene oxide (NGO) could be regarded as carrier to convey PS^54^ and build tumor‐targeted^55^ or photothermal treatment system.^56^ Interestingly, NGO would be preferable to separate from the PS in acid condition. Thus, Wu et al. designed a dual‐targeting (cellular targeting and subcellular targeting) nanosystem to control PDT.^57^ In this nanosystem, a cationic porphyrin was used as ^1^O_2_ PS, which is loaded onto the surface of the polyethylene glycol (PEG)‐functionalized and folic acid‐modified nanographene oxide (NGO‐based carrier for MitoTPP) through electrostatic interaction and π–π stacking (**Figure** **15**). PEG chains could enhance NGO's water dispersibility and biocompatibility; the folic acid moiety can specifically target the folate receptor‐positive cells. Under light irradiation, NGO can effectively inhibit the ^1^O_2_ generation until MitoTPP is released from its carrier in acidic environment (tumor cells).

**Figure 16:**
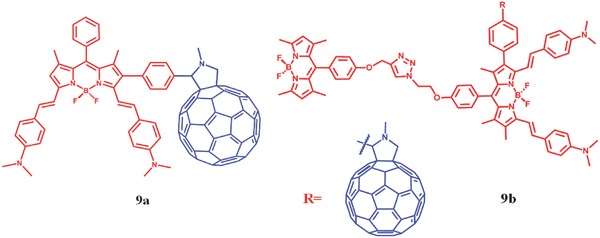
The structure of dimethylaminostyryl BODIPY‐C60 dyads and triads.

Instead of effect on the linker, the low pH could also influence the PS. In this case, the PSs are usually designed with amino groups (the H^+^ proton acceptor in acid condition).^58^ For example, combining dimethylaminostyryl BODIPY with C_60_, Zhao et al. proposed a method to switch the triplet excited states and ^1^O_2_ quantum yield based on FRET.^32^ The BODIPY moieties and C_60_ part are attached with each other by Prato reaction. **9a** is dyads (λ_abs_ = 627 nm, λ_em_ = 644 nm) while **9b** is triads (λ_abs_ = 502/627 nm, λ_em_ = 637 nm, **Figure** **16**).^32^ In this case, BODIPY moieties are FRET‐donor and light‐harvesting antenna, while C_60_ groups are FRET‐acceptor. The S_1_ state energy level of the BODIPY moieties in **9a** and **9b** are lower than C_60_, resulting in an inhibited FRET from BODIPY to C_60_. In addition, there is a lower charge transfer state which can quench triplet excited states. So little ^1^O_2_ could be generated in this condition. When dissolved in acid, the S_1_ state energy level of the BODIPY moieties would surpass the corresponding energy level of C_60_ due to the protonation of dimethylaminostyryl. At the same time, the energy level of charge transfer state is enhanced. So the triplet excited states and ^1^O_2_ could be produced. For **9a**, the ^1^O_2_ quantum yield is 1.1% in neutral condition and 26% in acid. For **9b**, that is 1.0%/1.4% in neutral and 22%/17% in acid, respectively.

**Figure 17:**
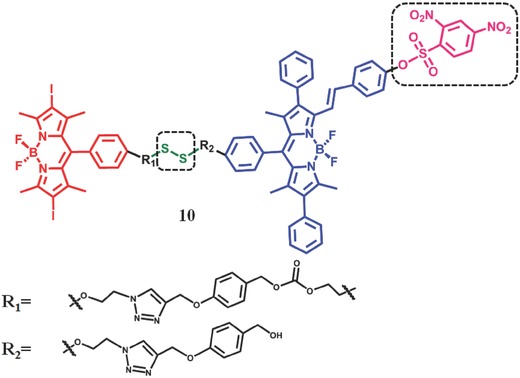
The structure of thiol‐activatable Bodipy–iodoBodipy dyads.

By integrating PDT with fluorescence imaging functionality in one molecule, theranostic is becoming a promising technology for the treatment of cancer.^59^ Zhao et al.^60^ reported a thiol‐activated difunctional‐PDT reagent **10** (**Figure** **17**) for theranostic purpose, in which the PDT effect and fluorescence imaging are implemented by different moieties, giving rise to both high ^1^O_2_ quantum yield and high fluorescence yield, simultaneously. The difunctional reagents are composed of PS, fluorophore (PL) and a disulfide bond (—S—S—), which can be cleaved by thiols such as Cys and GSH (Figure 17).^61^ The iodine‐BODIPY acts as PS and FRET‐donor, while BODIPY serves as fluorescence unit and the FRET‐acceptor. In the absence of thiols, the generation of ^1^O_2_ can be quenched by FRET from iodine‐BODIPY to BODIPY and the fluorescence of BODIPY can be inhibited by the 2,4‐dinitrobenzenesulfonate moieties (DNBS). That is the off state of ^1^O_2_ production and fluorescence. In the presence of thiols, FRET can be inhibited due to the cleavage of disulfide bond. So the efficiency of ISC is enhanced and the ^1^O_2_ can be produced with the irradiation of PS. Simultaneously, the red fluorescence of BODIPY was observed as a result of the decomposition of the DNBS group by the thiols. It turned out that the ^1^O_2_ quantum yield can be increased from 16.7%–71.5% and the fluorescence quantum yield can be enhanced from 1.3%–47.6%.

**Figure 18:**
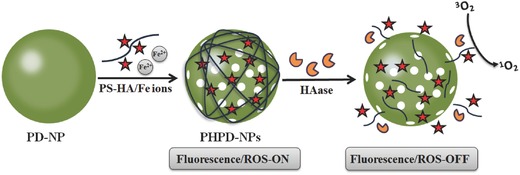
Synthetic routes of PS−hyaluronic acid conjugates shielded polydopamine nanoparticles (PHPD‐NPs) and its ^1^O_2_ on−off conversion. Adapted with permission.^62^ Copyright 2016, American Chemical Society.

Besides the disulfide cleavage, some cancer‐specific intracellular enzymes could also cut off the FRET process. Na et al. prepared core−shell nano PS consisting of polydopamine nanoparticle (PDNP) cores, surrounded by cancer‐specific PS‐conjugated hyaluronic acid (PS‐HA) shells (**Figure** **18**).^62^ PS‐HA play as the targeting moiety, while PDNP could quench the ^1^O_2_ generation though FRET. The cancer‐specific intracellular enzymes (e.g., hyaluronidase) is abundant in the tumor environment and plays key role for tumor proliferation and metastasis. This enzymes could separate the PS‐HA shell from PDNPs and recover the ^1^O_2_ generation ability of PS.

In fact, the key issues of controlling FRET effect are energy level matching and suitable linkers between the energy donor and the energy acceptor. As mentioned above, the overlapping extent of donor emission and acceptor absorption, namely their energy level matching, should be considered. Then, an appropriate linker should meet the following requirements: (1) bring the quencher and PS in close proximity, (2) being able to be selectively cleaved by specific factor (e.g. enzymes, pH). FRET‐modulation takes advantage of specificity‐linker to control the distance between donor and acceptor, resulting RET in on‐off switching effect. In the deactivation of excited PS, FRET and ISC compete with each other. If FRET surpass ISC, a low ^1^O_2_ quantum yield will be resulted; in contrast, once ISC is dominant, PS can be activated to produce ^1^O_2_.

### 3.2 Reversible FRET

In an interesting example, the generation of ^1^O_2_ can also be controlled upon the alterable direction of FRET. In this case, PS and FL are attached with each other, and there is an efficient excited energy transfer (EET) between PS and FL. Under certain conditions, excited energy transfers from PS to FL occurs, leading to the quench of PS, namely, no or little ^1^O_2_ is generated. However, under different condition, EET direction may be reversed, resulting in energy transfer from FL to PS, resulting in the activation of PS, therefore a high ^1^O_2_ quantum yield is detected.

**Figure 19:**
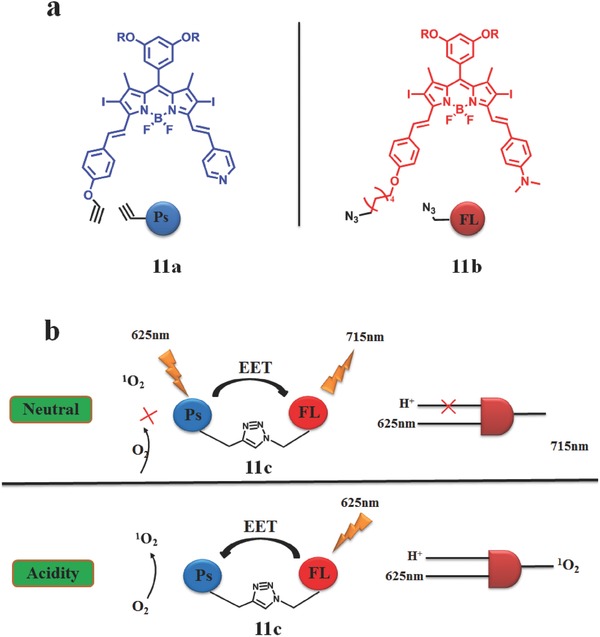
a) The chemical structure of PS and PL; b) working principle of DEMUX based on reversible FRET. Adapted with permission.^63^ Copyright 2013, The Royal Society of Chemistry.

Based on this mechanism, a very innovative experiment has been reported by Akkaya.^63^ A new strategy to control the on/off switching of ^1^O_2_ production by a molecular 1:2 demultiplexer (DEMUX)^64^ with FRET as the main mechanism. Actually, DEMUX is a kind of chemical logic gate^65^ and molecular logic gate,^66^ which can generate various output signals with one input in demand. These switches are controllable. Therefore, if reactive oxygen species and fluorescence were output signals, demultiplexer can be used to combine diagnosis and therapy into one unity for PDT. Following this logic, Akkaya et al. designed compound **11c** (**Figure** **19**) which was composed of two distyryl‐Bodipy modules connected together by Huisgen cycloaddition. One part of compound **11c** is a PS (**11a**) with two iodine atoms to enhance the ISC efficiency; another one is a PL (**11b**) with a sensitivity to proton (Figure 19).

When excited at 625 nm in a neutral environment, compound **11c** gives an intense emission band at 715 nm, which is an output of DEMUX. In this case, EET takes place from the PS to FL. While in acid addition, upon excited at 625 nm, EET direction reverse which takes place from FL to PS, resulting in the generation of ^1^O_2_ (Figure 19b). Actually, EET between PS and FL was a kind of FRET in this research. Generation of ^1^O_2_ was switch off/on with the acid driven of the EET direction. There is a promising application for compound **11c**: when transferred into a given region such as tumor cells, compound **11c** can efficiently generate ^1^O_2_ with fixed light irradiation for PDT, in the meantime, tumor cells and surrounding healthy tissues can be distinguished by different fluorescent intensity (healthy tissues are more brighter than tumor cells). No further biological researches were reported.

**Figure 20:**
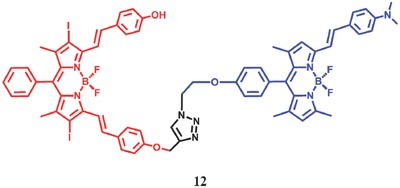
The structure of the IodoBodipy‐styrylBodipy dyads. Adapted with permission.^67^ Copyright 2014, American Chemical Society.

Zhao et al.^67^ also reported an interesting iodoBodipy‐styrylBodipy dyads **12** which can be used to switch on/off the generation of ^1^O_2_ based by the reversible FRET(**Figure** **20**). The PDT‐dyads are composed of the dimethylaminostyryl Bodipy moieties and the 4‐hydroxylphenyl Bodipy part. In order to enhance the ISC efficiency, iodine atoms were introduced into Bodipy; at the same time, the introduction of dimethylaminostyryl is to ensure the sensitivity of PDT‐dyads for acid. In the neutral condition, there is an effective FRET effect from hydroxylstyryl Bodipy group to dimethylaminostyryl Bodipy moieties, which can quench the fluorescence of the hydroxylstyryl Bodipy. In addition, the T_1_ of the hydroxyl styryl Bodipy part could be quenched by the excited energy transfer between hydroxyl styryl Bodipy group and dimethylaminostyryl Bodipy moieties. In this case, little ^1^O_2_ can be observed in this case (Φ_Δ_ = 0.06). In the presence of acid, the S_1_ and T_1_ state energy level of the dimethylamino styryl Bodipy group would be elevated, resulting in an inverse FRET effect from dimethylaminostyryl Bodipy moieties to hydroxylstyryl Bodipy group. Moreover, the formation of the charge transfer state is inhibited with the protonation of the dimethylamino group. So a long‐lived T_1_ state (τ_T_ = 1.6 µs) and high‐efficiency ^1^O_2_ quantum yield (Φ_Δ_ = 0.59) were observed for **12**. In should be noted that the photophysical properties of this dyads can be recovered completely with the acid neutralized by base.

**Figure 21:**
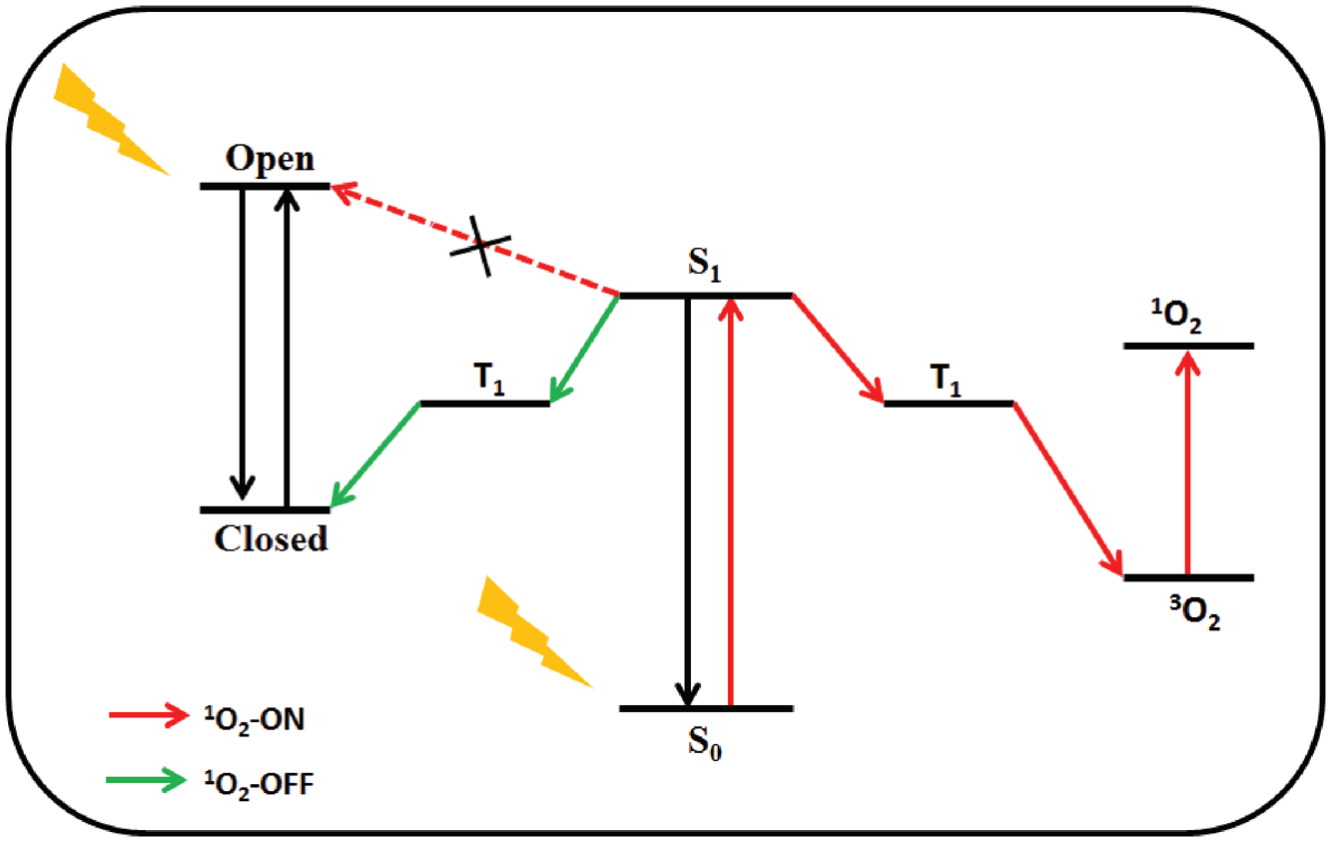
Common schematic representationof photocontrol ^1^O_2_ on‐off state.

Notably, the critical factor in regulating FRET is how to change the donor and acceptor energy level by external stimulus. As mentioned above, various chemical stimuli (such as pH, enzyme) have been employed to modulate the properties of PS, resulting in on/off or high/low ^1^O_2_ quantum yield. In these systems, however, there are still some drawbacks limiting their application in PDT, for instance: (1) some control conditions is beyond the normal physiological conditions,^68^ (2) control strategy such as enzymatic‐cleavable FRET linkers or pH‐manipulative PET are irreversible, namely, they cannot recover its original state. Herein, it is imperative to design new strategy for regulating ^1^O_2_ on‐off reversibly. Concerning this aspect, some compounds such as diarylethene derivatives can interconvert between the open forms (with high energy levels) and closed forms (with low energy levels) upon irradiation at different wavelength,^69^ which enable reversible FRET process. Thus, excited state of PS can be quenched by the energy transfer from PS to the closed forms of diarylethene, thus the production of ^1^O_2_ is inhibited. Conversely, ^1^O_2_ can be easily generated when the energy transfer between PS and the open forms of diarylethene is inhibitory (**Figure** **21**).

**Figure 22:**
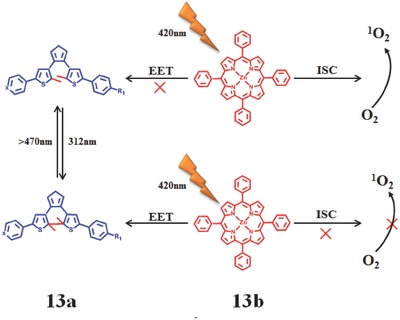
Controllable generation of ^1^O_2_ based on photochemical switches of diarylethene. PS is shown in red and diarylethene is shown in blue. Adapted with permission.^70^ Copyright 2014, American Chemical Society.

Feringa et al.^70^ designed a novel bicomponent system to modulate the generation of ^1^O_2_ with photoirradiation as control (**Figure** **22**). The bicomponent system are made up of diarylethene (photochromic switch, **13a**) and Zinc−tetraphenylporphyrin (PS, **13b**, Φ_Δ_ = 0.84).^71^ When irradiated at UV and visible light, diarylethene molecular can interconvert between their colorless open and colored closed forms, respectively.^72^ The molecular geometry and triplet energy levels of the open and closed forms of diarylethene are different. When diarylethene transforms into closed form with irradiation at 314 nm, triplet state energy transfer from **13b** (excited by 420 nm) to the closed form of the diarylethene suppresses the ^1^O_2_ production; in contrast, the open form of diarylethene is dominant when irradiated by visible light (>470 nm). In this case, no triplet state energy transfer occurs and the ^1^O_2_ can be generated effectively. The whole process of modulation is reversible. Later, this FRET mechanism was develeped into supramolecular assemblies and MOF.

**Figure 23:**
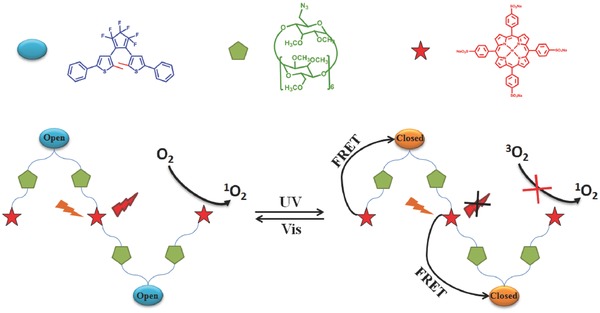
Schematic representation for photo‐controlling ^1^O_2_ on‐off based on FRET conversion. Adapted with permission.^73^ Copyright 2016, The Royal Society of Chemistry.

Liu et al. apply this reversible energy transfer mechanism into photo controlled supramolecular assemblies (**Figure** **23**).^73^ Dithienylethene‐modified permethylated β‐cyclodextrins and porphyrin derivatives forms assemblies in water through strong binding. Porphyrin is energy‐donor and dithienylethene acts as energy‐acceptor. Compared with other porphyrin/dithienylethene systems, this supramolecular assemblies system is with good water solubility and biocompatibility due to the introduction of permethyl‐β‐cyclodextrin. In addition, there is a high FRET efficiency (93.4%) between porphyrin and dithienylethene groups. The response of switch on/off for ^1^O_2_ generation is fast (switch on‐off: 50 s; switch off‐on: 120 s).

**Figure 24:**
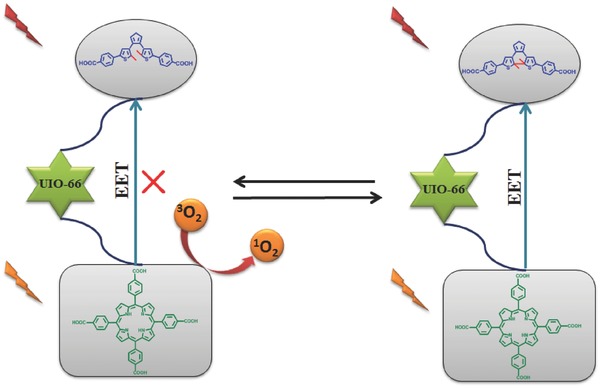
Defective UiO‐66 with inserted porphyrin and diarylethene and their photoregulation ^1^O_2_ generation process.

Instead of supramolecular assemble system, Zhou et al. firstly developed this reversible FRET mechanism into MOF system for the photooxidation of 1,5‐dihydroxynaphthalene.^74^ Then, they build another photosensitizing MOF system with UiO‐66, porphyrin PSs and dithienylethene derivatives (fluorescent molecular switch) (**Figure** **24**).^75^ UiO‐66 was regarded as a nanoplatform to storage and transport PDT agents. More importantly, it is possible to optimize the controllability of PDT performance by carefully modulating the ratios between PS and dithienylethene switch. In addition, without linkers between them, FRET happened efficiently in this MOF microenvironment, it successfully realize a reversible switch on/off of ^1^O_2_ generation for PDT.

## 4 ICT Regulation Mechanism

ICT has been widely applied in many fields such as fluorescence probe,^76^ fluorescent chemosensor,^77^ etc.^78^ By modulating the properties of the electron donor and acceptor, it is possible to change the efficiency of ICT. ICT can intensely impact the fluorescence and ^1^O_2_ quantum yields via competing with other deactivation processes such as ISC and PET. There are many studies of the effect of the different donor and acceptor on ICT efficiency,^79^ but little was known about the modulation of ^1^O_2_ generation with ICT variation.

**Figure 25:**
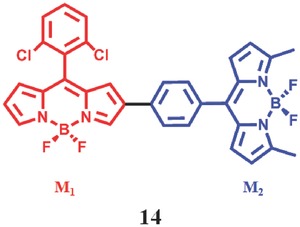
The structure of BODIPY dimer. Both M_1_ and M_2_ are BODIPY monomer. Reproduced with permission.^80^ Copyright 2013, American Chemical Society.

Yang et al.^80^ prepared a BODIPY dimer (**14**) and investigated its mechanism of ^1^O_2_ generation (**Figure** **25**). It is interesting that the ^1^O_2_ producing ability of this BODIPY dimer can change in non‐polar/polar microenvironments. Only in low polar solvent (such as hexane, cyclohexane and toluene) can ^1^O_2_ be generated significantly while no or little ^1^O_2_ can be produced in polar solvent. Triplet excited state (τ_T_ = 49 µs in argon‐saturated hexane) only exists in BODIPY dimer rather than in BODIPY monomer, which is demonstrated by transient absorption spectra.^81^ In fact, there is a significant ICT effect in polar solvents which can compete with ISC, resulting in quenching of the ^1^O_2_ production.

**Figure 26:**
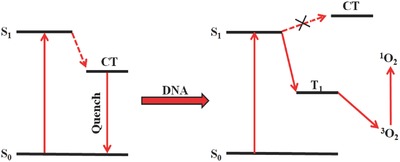
Schematic representation for DNA regulated ^1^O_2_ generation based on ICT energy‐level variation.

Hirakawa et al. designed meso‐(9‐anthryl) tris(N‐methyl‐p‐pyridinio) porphyrin, which is composed of anthracene (D) and porphyrin (A).^82^ In this PS, the intramolecular electron transfer from the anthracene moiety to the porphyrin moiety forms a CT state, providing a fast deactivation pathway instead of producing ^1^O_2_. Unlike previous pH modulation, the anionic DNA were used to interact with cationic porphyrin, and it inhibits the electron transfer quenching via rising the CT state energy level (**Figure** **26**). The ^1^O_2_ quantum yield increased to 0.22, no ^1^O_2_ production was detected in the absence of DNA. Thus, the electron‐donor connecting TMPyP type PS could control the photosensitized ^1^O_2_ generation through this ICT mechanism.

**Figure 27:**
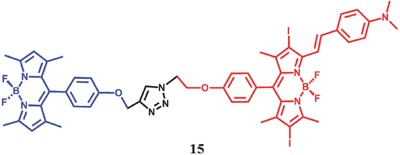
The structure of the dimethylaminostyryl iodoBodipy‐Bodipy dyads. Adapted with permission.^67^ Copyright 2014, American Chemical Society.

Actually, ICT can compete with other processes as well, such as PET. Zhao et al.^67^ proposed a strategy to modulate the ^1^O_2_ generation by mainly controlling the ICT effect of PS. In this dyads **15**, the dimethylaminostyryl iodo‐Bodipy and the unsubstituted Bodipy are connected with each other (**Figure** **27**). In the absence of acid, there is an effective FRET effect from unsubstituted Bodipy to dimethylaminostyryl iodo‐Bodipy. However, the S_1_ and T_1_ of iodo‐Bodipy can be quenched by the ICT effect, resulting in a low quantum yield of ^1^O_2_ (Φ_Δ_ = 0.07). With the addition of acid, the ICT effect disappeared with the protonation of dimethylaminostyryl group, resulting in a long‐lived dimethylaminostyryl (τ_T_ = 3.1 µs) and a high ^1^O_2_ quantum yield (Φ_Δ_ = 0.74). Again, the photophysical properties of this dyads can be recovered completely after the addition of base.

## 5 The Competition between EET and PET

**Figure 28:**
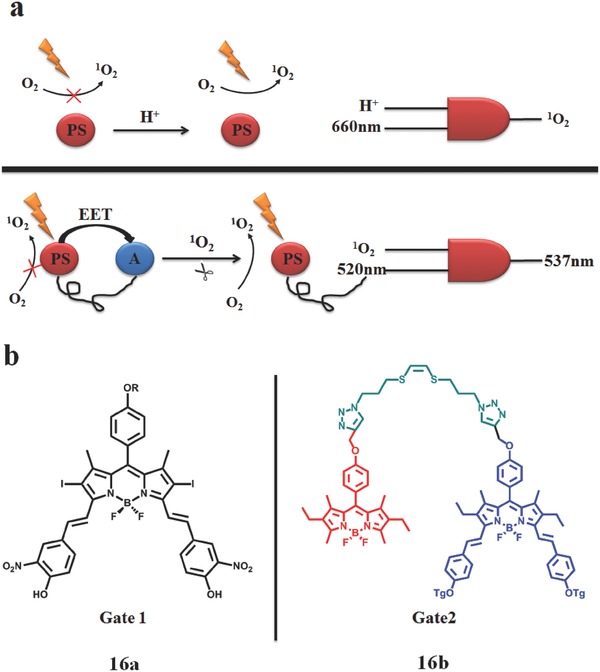
a) Operational mechanism for the AND logic gates. Top: modulation of ^1^O_2_ ; bottom: fluorescence self‐reporting. b) The structure of Gate1 and Gate2. In Gate2, the donor of EET are shown in red part while blue part represents EET acceptor. Adapted with permission.^84^

In the generation of ^1^O_2_, there are always several different mechanisms competing with each other, e.g. FRET, PET and ICT. They are mainly from the competition between energy transfer (EET) and electron transfer (PET). Derived from these competition, the emergence of molecular logic gates, emerged to finely tune the ^1^O_2_ generation. Molecular logic gates, as a promising strategy,^83^ was rarely applied to PDT. Combining molecular logic gates with PDT, Akkaya et al.^84^ reported a controllable and self‐reporting PDT, realized by series logic gate. The cascading of molecular logic gates are composed of two AND logic operation (**Figure** **28**). In the first logic gates, light (660 nm) and acidic environment are input signal, ^1^O_2_ is the output. BODIPY connected with two nitro (p*K*_a_ = 6.92) is responsible for Gate 1 ( **16a** ). Only in the presence of acid can ^1^O_2_ be generated as a result of the protonation of Gate 1. There is no related interpretation mechanism for pH‐controllable PDT. For the second logic gates, ^1^O_2_ (the output of gate1) and 520 nm light are the input, the output is 537 nm light. Gate 2 is composed of an EET donor (D) and acceptor (A) portion which are connected to each other by a ^1^O_2_‐cleavable linker ((*Z*)‐1,2‐bis(alkylthio) ethane, **16b**). In the absence of ^1^O_2_, there is an effective excitation energy transfer (85.0%) between D and A, shutting down the production of ^1^O_2_. In contrast, D can emit at 537 nm in the present of ^1^O_2_ which can break the linker. Gates 1 and 2 were embedded together into a micelle for a continuous logic operation. When this logic gates are introduced into body, controllable‐PDT efficiency can be revealed by the fluorescence intensity at 537 nm.

## 6 Factitious Captivity and Release

### 6.1 Factitious Captivity and Release of PSs

Besides regulating the PSs themselves via PET, FRET or ICT, there is another direct regulation method — captivity and release of PS or singlet oxygen. In this part, the ^1^O_2_ PS is usually confined in the cage of core or shell part (e.g. nanoparticles, micelle or self‐assembly), which are sensitive to the special environment of cancer cell or tumor (e.g. low pH, high concentration of GSH). When the modified PSs comes to the special tumor environment, the active PSs could be released from the cage and generate ^1^O_2_. Some typical stimulated examples are listed below.

**Figure 29:**
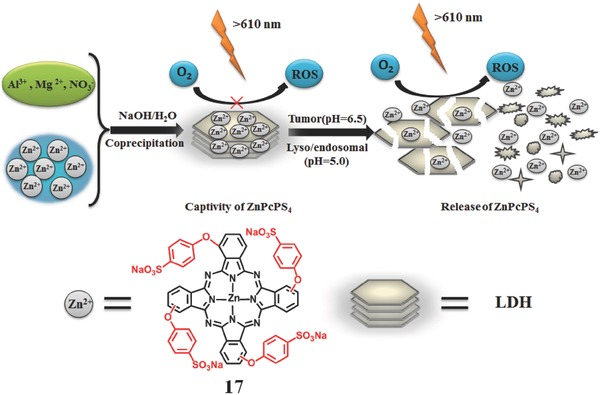
The synthetic route of LDH–ZnPcPS_4_ and its working principle for controllable PDT. Adapted with permission.^85^

Huang et al.^85^ provided an interesting and provident investigation about switching on/off the generation of ^1^O_2_ by a layered double hydroxide (LDH) with the pH‐responsive property. Herein, zinc (II) phthalocyanine substituted with 4‐sulfonatophenoxy groups (ZnPcPS4, **17**) combined with LDH via electrostatic interaction was synthesized (**Figure** **29**).^85^ When there is no acid, LDH–ZnPcPS4 has little capacity to generate ^1^O_2_ due to the stable structure of nanohybrid. However, in slightly acidic media (pH = 6.5 or 5.0), ZnPcPS4 can be efficiently released from the LDH carrier due to the collapse of the LDH structure, resulting in recovery of the photoactivities.

**Figure 30:**
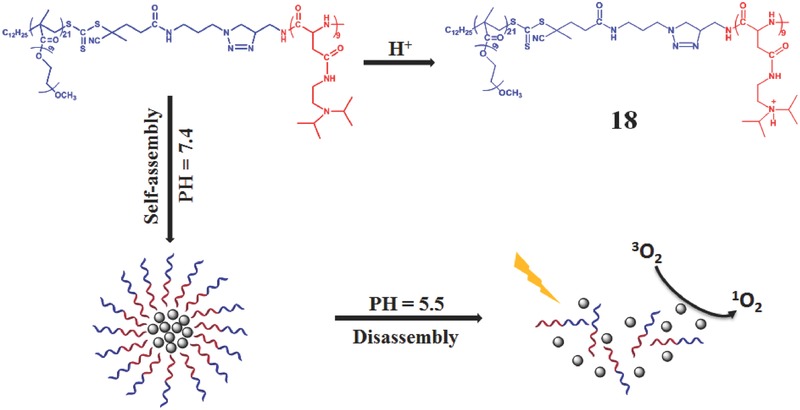
Structure of multifunctional polypeptide micelle and its pH‐responsive PDT process. Adapted with permission.^58^ Copyright 2016, American Chemical Society.

Liu et al. reported a pH‐responsive multifunctional polypeptide micelle for simultaneous imaging and in vitro PDT (**Figure** **30**).^58^ The BODIPY‐Br_2_ was used for the efficient ^1^O_2_ generation. By hydrophobic interaction, BODIPY‐Br_2_ connected with amphiphilic copolypeptide micelles via a ring‐opening polymerization and click reaction. It is worth to mention that the diisopropylethylamine groups conjugate to the polypeptide side chains. Under normal microenvironment, it forms the hydrophobic core of BODIPY‐Br_2_ and hydrophilic shell for improving the solubility and stabilization. However, at lower pH such as 5.5, the diisopropylethylamine groups could be protonated **18**, and its role converts from the hydrophobicity to hydrophilicity. Subsequently, the core−shell structured micelle degrade and it could release BODIPY‐Br_2_ as shown in Figure 30.

**Figure 31:**
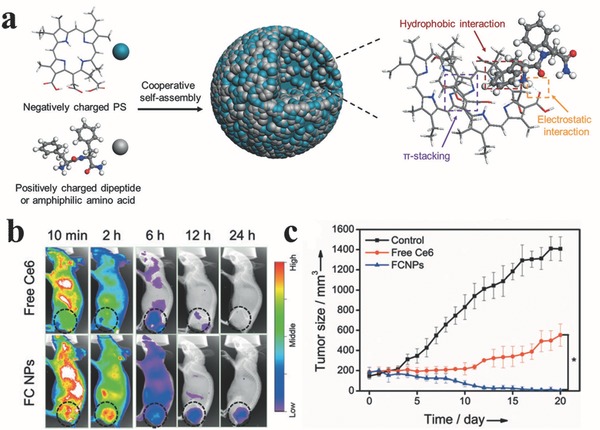
a) synthetic route of photosensitive nanoparticles based on amphiphilic dipeptide‐tuned self‐assembly. b) The fluorescence images of MCF7‐tumor‐bearing nude mice through a tail intravenous injection with FCNPs and free Ce6 (equivalent Ce6 4.0 mg kg^–1^ body) at different times. tumor sites were shown in black circles. c) tumor growth test within 20 days. Reproduced with permission.^87^

Based on the peptide‐ and protein‐Based assembly, Yan et al. develop a new type of delivery carriers for regulating photosensitization and enhancing PDT effects.^86^ Compared with the free PSs, these assembly show enhanced PDT effect after the PSs released from these assembly. For example, 9‐Fluorenylmethoxycarbonyl‐1‐lysine (an amphiphilic amino acid with antiinflammatory activity) and Chlorin e6 (Ce6, a model hydrophobic photosensitive drug) formed the self‐assembly (FCNPs) through electrostatic force, π–π stacking and hydrophobic interactions (**Figure** **31**a).^87^ These self‐assembly could be stimulated by pH, surfactant and enzyme as they are on‐demand to release drug in tumors. The fluorescence intensity of Ce6 could reflect the the distribution and the concentration of FCNPs. The in vivo biodistribution showed that FCNP has a selective accumulation in the tumor (Figure 31b). Compared wtih free Ce6, the PCNPs exhibit sustained Ce6 fluorescence at the tumor site for 24 h. Due to this retention of photosensitive drugs in the tumor tissue, the tumors of FCNP‐treated mice were suppressd and almost completely eradicated during the PDT (Figure 31c).

**Figure 32:**
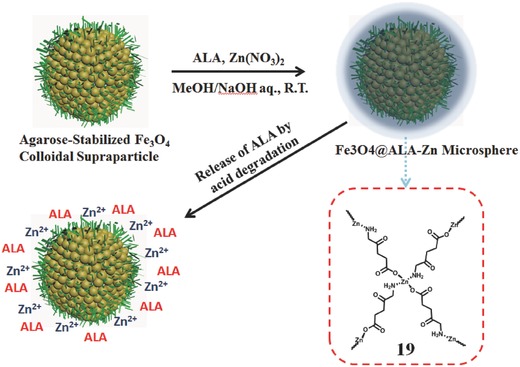
Preparation process of Fe_3_O_4_@ALA‐ZnII microsphere and the pH‐sensitive release of PS. Adapted with permission.^88^

Instead of the most PS in the core part, Guo et al. confined the pH sensitive PS, 5‐aminolevulinic acid Zn(II) coordination polymer (ALA‐Zn^II^, **19**) in the shell part, and magnetite colloidal supraparticles (Fe_3_O_4_) in the core part (**Figure** **32**).^88^ ALA could selectively accumulate in tumorous tissues and be metalized to the PS PpIX for producing ^1^O_2_ after the visible light irradiation. In this PS (Fe_3_O_4_@ALA‐Zn^II^), the ALA release could be triggered by a small pH variation (pH 7.4 to 6.0 and 5.0). Compared with ALA, Fe_3_O_4_@ALA‐Zn^II^ has better PDT activity for T24 cells, and show good biocompatibility with the normal 293T cells.

**Figure 33:**
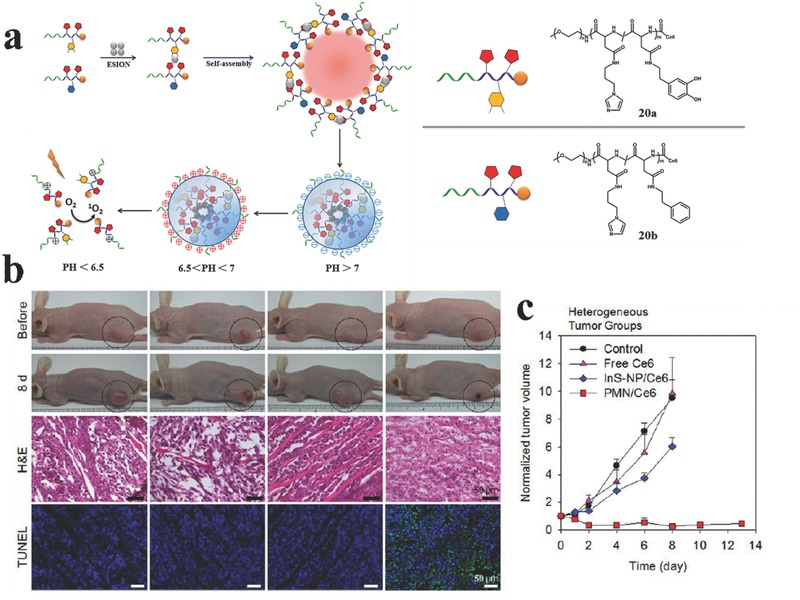
a) Synthetic route of pH‐responsive Magnetic Nanogrenades and their photoactivity change in different pH levels. b) Upper: photograph of mice bearing heterogeneous tumors before and after PDT. Below: H&E and TUNEL staining of tumor tissue sections (c) Measured heterogeneous tumor volumes in the four treatment groups for 10 days. Reproduced with permission.^89^ Copyright 2014, American Chemical Society.

The microenvironment of tumors is usually with a pH of ca. 6.8, and endo/lysosomes has even pH values of 5.0–5.5. Without any targeting agents, Hyeon et al.^89^ reported the tumor pH‐sensitive magnetic nanogrenades (PMNs, **Figure** **33**), which is composed of self‐assembled iron oxide nanoparticles (the core part for T_1_ MRI contrast agent) and pH‐responsive ligands (the shell part by incorporating ionizable moieties on the polymeric ligand **20a, 20b**). In this shell part, it involves a two‐stage pH activation: (1) for increased cell adsorption and permeation at the tumor microenvironment, imidazole (p*K*a, ≈6.8) incorporated could impart pH sensitivity; (2) for releasing the diagnosis and PDT agents under tumor endo/lysosomal pH of ≈5.5, 3‐phenyl‐1‐propylamine incorporated could produce a critical phase transition of PMNs, and destroy the self‐assembly structure. The therapeutic effect of PMNs in tumors of more heterogeneous and drug‐resistant nature were further demonstrated. Compared with pH‐Insensitive Nanoparticle Assemblies (InS‐NPs) and free Ce6, PMNs produced great tumor growth inhibition (Figure 33b, c). In the PMN treated group, many cells in the tumor tissue and microvasculature as well as fibroblasts showed considerable destruction. Therefore, this pH sensitivity plays an important role in the improved anticancer therapeutic efficacy of PMNs.

### 6.2 Chemical Control for ^1^O_2_ Release and Capture

Instead of the above mentioned methods of capture and release of the PSs, ^1^O_2_ could also be reversibly restored by chemical method in some cases. ^1^O_2_ could reversibly react with some specific polycyclic aromatic hydrocarbons (e.g. naphthalene, anthracene or pyridine derivatives),^90^ and form endoperoxide which can subsequent release ^1^O_2_ via warming and revert to the original state. However, tumor hypoxia^91^ and PDT‐induced hypoxia^92^ hinder the development of PDT. Intermittent irradiation is supposed for recovery of intracellular oxygen. To some extent, this chemical controlled ^1^O_2_ strategy are supposed to relieve the deficiency of oxygen problem in PDT.

**Figure 34:**
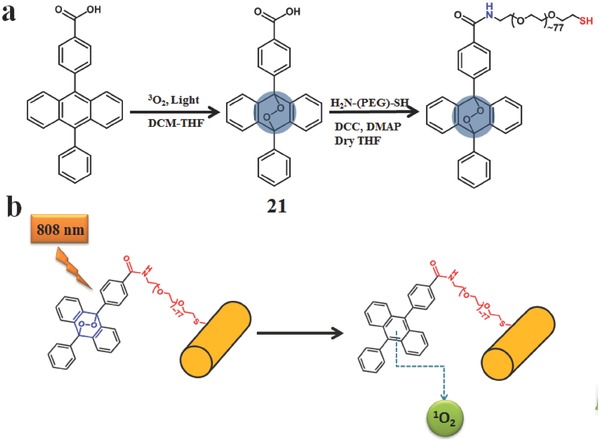
a) Synthetic route of anthracene endoperoxide derivative and its further functionalization with gold nanorod; b) the release of singlet oxygen due to thermal cycloreversion of the endoperoxides upon plasmonic heating gold nanorods. Adapted with permission.^93^

Anthracene is a good substrate to form stable endoperoxides at ambient temperature (or 37 °C), but these endoperoxides could rapidly and completely come to cycloreversion after heating. Based on this method, Yoon and Akkaya et al. make good use of plasmonic heating of gold nanorods for remote‐control ^1^O_2_ release.^93^ In this PS, the anthracene derivative was firstly oxidized into endoperoxides **21** by the MB under irradiation in the presence of oxygen (**Figure** **34**). Then amine‐PEG‐thiol coupling with endoperoxides used to increase water solubility and to target at the gold nanorods for the convenient thermal transport. When this PS irradiated with a laser with 830 nm at room temperature, the absorption intensity of singlet‐oxygen trap (1,3‐diphenylbenzofuran) at 414 nm decrease, indicating the generation of the singlet oxygen.

**Figure 35:**
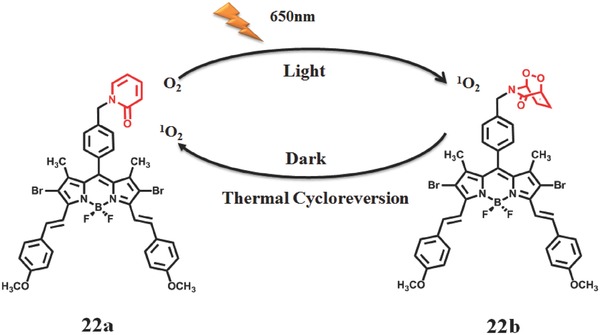
The cycle diagram of continuous release of ^1^O_2_ based on bifunctional BODIPY derivative PS. Adapted from with permission.^94^

Later, Akkaya et al.^94^ prepared a bifunctional PS composed of 2,6‐dibromo distyryl BODIPY and 2‐pyridone (**Figure** **35**). 2,6‐dibromo distyryl BODIPY is a high‐efficiency PS with long‐wavelength absorption; the endoperoxides of 2‐pyridone and derivatives were reported to generate ^1^O_2_ efficiently by clean (no side reactions) cycloreversion reactions. The circulating principle of this system is described as follows: when irradiated at 650 nm, compound **22a** is excited to produce ^1^O_2_ by photosensitive process and a part of ^1^O_2_ is stored in endoperoxide of 2‐pyridone (**22b**); in contrast, ^1^O_2_ can be released with the thermal cycloreversion of compound **22b** in the absence of light, compound **22a** is recovered at the same time. Obviously, this process is reversible.

**Figure 36:**
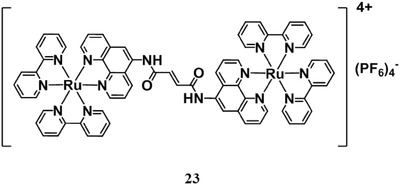
The structure of the binuclear ruthenium(II) dimer. Reproduced with permission.^95^ Copyright 2014, The Royal Society of Chemistry.

There are also other methods appeared. For example, Lovell and his co‐workers prepared a binuclear ruthenium(II) dimer **23** which is bridged by an alkene linker (**Figure** **36**).^95^ As the alkene linker is cleaved by MB with red‐laser irradiation, there is an intermediate chemical amplification effect on the ^1^O_2_ generation after the subsequent blue‐laser irradiation. It is different from previous ^1^O_2_ quenching effect. With the further development of photochemistry and photophysics, these methods could also built up their own mechanisms in the future.

## 7 Conclusions and Outlook

In this review, we have summarized the recent developments in controllable photodynamic therapy and some novel strategies of designing activatable PSs. We introduced various regulation methods to control the ^1^O_2_ production, such as PET, FRET, ICT, etc. These mechanisms can be employed to modulate the generation of ^1^O_2_. It should be noted that the crucial concept of controllable PDT reagents is to selectively generate ^1^O_2_ in tumor tissues and to avoid damaging normal tissues and organs. Based on PET mechanism, supramolecular photonic therapeutic reagents, environment‐sensitive PSs and BODIPY derivatives have been reported, achieving ^1^O_2_ on/off by pH or polarity variation. Amino protonation is the most used strategy to regulate PET process, and it is necessary to develop new method for effective PET switch. Controlling of FRET is one of the most common mechanism to modulate ^1^O_2_ production. The key part of inhibitive FRET is to find a suitable linker, which can be cleaved by tumor‐associated specific conditions such as lower pH and overexpression proteins or enzymes. Except for a cleaveable linker, using nano materials as carrier, combining PS and quencher into a single form is also effective to achieve switching of ^1^O_2_ production. By regulating the energy level of FRET‐acceptor, the direction of FRET can be inverted, resulting in an activation and deactivation of PS, respectively. Reversible FRET is becoming a promising strategy to modulate PDT. In addition, the using of photoconversion molecules and molecular logic‐gates is also promising. In a word, by efficient FRET to quench PS, by prohibitive FRET to reactivate PS. Upon competing with ISC directly, ICT can effectively restrain the generation of ^1^O_2_. The charge‐transfer (CT) state of donor and acceptor can be influenced by solvent polarity and pH. However, ICT was rarely used to modulate PDT. There is still much room to apply ICT in activatable PDT.

Except for using various energy and electron transfer mechanism, physical confinement and controllable release of PS is also an important way to regulate PDT. As for this strategy, PS are always coated by some tumor targetable materials such as LDH or micelles, which can be decomposed only in tumor. In addition, the materials act as carrier and provide platform for incorporating many other functional molecules, achieving multiple treatment (such as photothermal therapy) and diagnose.

Tumor hypoxia and PDT‐induced hypoxia seriously hinder the development of PDT. Therefore it is necessary to complement oxygen timely. Fortunately, ^1^O_2_ can also be generated in dark due to the thermal conversion of endoperoxide, that is ‘chemical control for ^1^O_2_ release and capture’. We believed this strategy is promising.

Compared with traditional PDT reagents, which are untargetable, controllable PSs opens new avenue for PDT in clinical practice by selective control of photosensitization. However, some conditions required for controllable PDT have surpassed the normal physiological condition and some strategies are complicated or intricate. In addition, sometimes controllable PDT and high ^1^O_2_ quantum yield cannot be achived simultaneously. Hence, it should be realized that even more wonderful strategies or PS should be designed to achieve more efficient and safer PDT.

## Conflict of Interest

The authors declare no conflict of interest.

## References

[1] a) J. P. Celli , B. Q. Spring , I. Rizvi , C. L. Evans , K. S. Samkoe , S. Verma , B. W. Pogue , T. Hasan , Chem. Rev. 2010, 110, 2795; 20353192 10.1021/cr900300p PMC2896821

[2] a) L. Brannon‐Peppas , J. O. Blanchette , Adv. Drug. Deliver. Rev. 2012, 64, 206; 10.1016/j.addr.2004.02.014 15350294

[3] a) P. Agostinis , K. Berg , K. A. Cengel , T. H. Foster , A. W. Girotti , S. O. Gollnick , S. M. Hahn , M. R. Hamblin , A. Juzeniene , D. Kessel , Ca‐Cancer J. Clin. 2011, 61, 250; 21617154 10.3322/caac.20114 PMC3209659

[4] a) V. Bogoeva , M. Siksjo , K. G. Saeterbo , T. B. Melo , A. Bjorkoy , M. Lindgren , O. A. Gederaas , Photodiagnosis Photodyn. Ther. 2016, 14, 9; 26845686 10.1016/j.pdpdt.2016.01.012

[5] a) N. J. Turro , V. Ramamurthy , J. C. Scaiano , Principles of molecular photochemistry: an introduction, University Science Books, Sausalito, California, USA 2009;

[6] a) N. Adarsh , R. R. Avirah , D. Ramaiah , Org. Lett. 2010, 12, 5720; 21090576 10.1021/ol102562k

[7] J. Zhao , W. Wu , J. Sun , S. Guo , Chem. Soc. Rev. 2013, 42, 5323. 23450221 10.1039/c3cs35531d

[8] a) Y. Liu , Y. Liu , W. Bu , C. Cheng , C. Zuo , Q. Xiao , Y. Sun , D. Ni , C. Zhang , J. Liu , Angew. Chem. Int. Ed. 2015, 54, 8105; 10.1002/anie.201500478 26012928

[9] a) K. Plaetzer , B. Krammer , J. Berlanda , F. Berr , T. Kiesslich , Lasers Med. Sci. 2009, 24, 259; 18247081 10.1007/s10103-008-0539-1

[10] a) A. E. O'Connor , W. M. Gallagher , A. T. Byrne , Photochem. Photobiol. 2009, 85, 1053; 19682322 10.1111/j.1751-1097.2009.00585.x

[11] P. B. van Driel , M. C. Boonstra , M. D. Slooter , R. Heukers , M. A. Stammes , T. J. Snoeks , H. S. de Bruijn , P. J. van Diest , A. L. Vahrmeijer , P. M. v. B. en Henegouwen , J. Control. Release 2016, 229, 93. 26988602 10.1016/j.jconrel.2016.03.014 PMC7116242

[12] J. Eichler , J. Knof , H. Lenz , Radiat. Environ. Biophys. 1977, 14, 239. 928630 10.1007/BF01323942

[13] a) J. Zhao , S. Ji , W. Wu , W. Wu , H. Guo , J. Sun , H. Sun , Y. Liu , Q. Li , L. Huang , RSC Adv. 2012, 2, 1712;

[14] a) J. F. Lovell , T. W. Liu , J. Chen , G. Zheng , Chem. Rev. 2010, 110, 2839; 20104890 10.1021/cr900236h

[15] a) X.‐F. Zhang , W. Guo , J. Photochem. Photobiol. A 2011, 225, 117;

[16] J. Fan , M. Hu , P. Zhan , X. Peng , Chem. Soc. Rev. 2013, 42, 29. 23059554 10.1039/c2cs35273g

[17] Y. Urano , D. Asanuma , Y. Hama , Y. Koyama , T. Barrett , M. Kamiya , T. Nagano , T. Watanabe , A. Hasegawa , P. L. Choyke , Nat. Med. 2009, 15, 104. 19029979 10.1038/nm.1854 PMC2790281

[18] M. Abbas , Q. Zou , S. Li , X. Yan , Adv. Mater. 2017, 29, 1605021. 10.1002/adma.201605021 28060418

[19] a) T. Miura , Y. Urano , K. Tanaka , T. Nagano , K. Ohkubo , S. Fukuzumi , J. Am. Chem. Soc. 2003, 125, 8666; 12848574 10.1021/ja035282s

[20] X.‐F. Zhang , J. Fluoresc. 2011, 21, 1559. 21264499 10.1007/s10895-011-0844-0

[21] a) M. E. Daraio , P. F. Aramendía , E. San Román , Chem. Phys. Lett. 1996, 250, 203;

[22] a) K. Velmurugan , S. Suresh , S. Santhoshkumar , M. Saranya , R. Nandhakumar , Luminescence 2016, 31, 722; 26333533 10.1002/bio.3016

[23] S. O. McDonnell , M. J. Hall , L. T. Allen , A. Byrne , W. M. Gallagher , D. F. O'shea , J. Am. Chem. Soc. 2005, 127, 16360. 16305199 10.1021/ja0553497

[24] T. Yogo , Y. Urano , A. Mizushima , H. Sunahara , T. Inoue , K. Hirose , M. Iino , K. Kikuchi , T. Nagano , Proc. Natl. Acad. Sci. USA 2008, 105, 28. 18172220 10.1073/pnas.0611717105 PMC2224201

[25] T. Inoue , K. Kikuchi , K. Hirose , M. Iino , T. Nagano , Biorg. Med. Chem. Lett. 1999, 9, 1697. 10.1016/s0960-894x(99)00256-5 10397504

[26] W. Nakanishi , K. Kikuchi , T. Inoue , K. Hirose , M. Iino , T. Nagano , Biorg. Med. Chem. Lett. 2002, 12, 911. 10.1016/s0960-894x(02)00044-6 11958992

[27] a) Ł. Lamch , W. Tylus , M. Jewgiński , R. Latajka , K. A. Wilk , J. Phys. Chem. B 2016, 120, 12768; 27973818 10.1021/acs.jpcb.6b10267

[28] S. Ozlem , E. U. Akkaya , J. Am. Chem. Soc. 2008, 131, 48. 10.1021/ja808389t 19086786

[29] U. Pischel , Angew. Chem. Int. Ed. 2007, 46, 4026. 10.1002/anie.200603990 17385771

[30] P. Montcourrier , P. Mangeat , C. Valembois , G. Salazar , A. Sahuquet , C. Duperray , H. Rochefort , J. Cell Sci. 1994, 107, 2381. 7844158 10.1242/jcs.107.9.2381

[31] I. Cameron , N. Smith , T. Pool , R. Sparks , Cancer Res. 1980, 40, 1493. 7370987

[32] L. Huang , J. Zhao , J. Mater. Chem. C 2015, 3, 538.

[33] J. Szöllosi , S. Damjanovich , L. Mátyus , Cytometry 1998, 34, 159. 9725457

[34] R. B. Sekar , A. Periasamy , J. Cell Biol. 2003, 160, 629. 12615908 10.1083/jcb.200210140 PMC2173363

[35] E.‐Y. Chuang , C.‐C. Lin , K.‐J. Chen , D.‐H. Wan , K.‐J. Lin , Y.‐C. Ho , P.‐Y. Lin , H.‐W. Sung , Biomaterials 2016, 93, 48. 27070992 10.1016/j.biomaterials.2016.03.040

[36] J. C. Qin , J. Yan , B. d. Wang , Z. y. Yang , Tetrahedron Lett. 2016, 57, 1935.

[37] V. Miskolci , B. Wu , Y. Moshfegh , D. Cox , L. Hodgson , J. Immunol. 2016, 196, 3479. 26951800 10.4049/jimmunol.1501655 PMC4821714

[38] a) M. Kotresh , K. Adarsh , M. Shivkumar , B. Mulimani , M. Savadatti , S. Inamdar , Luminescence 2015, 31, 760; 26333828 10.1002/bio.3021

[39] Z. Bai , Y. Liu , P. Zhang , J. Guo , Y. Ma , X. Yun , X. Zhao , R. Zhong , F. Zhang , Luminesc. 2015, 31, 688. 10.1002/bio.3012 27037968

[40] D. L. Dexter , J. Chem. Phys. 1953, 21, 836.

[41] C. G. Dos Remedios , P. D. Moens , J. Struct. Biol. 1995, 115, 175. 7577238 10.1006/jsbi.1995.1042

[42] J. F. Lovell , J. Chen , M. T. Jarvi , W. G. Cao , A. D. Allen , Y. Liu , T. T. Tidwell , B. C. Wilson , G. Zheng , J. Phys. Chem. B 2015, 113, 3203. 10.1021/jp810324v 19708269

[43] a) N. S. James , P. Joshi , T. Y. Ohulchanskyy , Y. Chen , W. Tabaczynski , F. Durrani , M. Shibata , R. K. Pandey , Eur. J. Med. Chem. 2016, 122, 770; 27543778 10.1016/j.ejmech.2016.06.045 PMC5720162

[44] a) P. C. Ray , Z. Fan , R. A. Crouch , S. S. Sinha , A. Pramanik , Chem. Soc. Rev. 2014, 43, 6370; 24902784 10.1039/c3cs60476d

[45] a) M. Verhille , H. Benachour , A. Ibrahim , M. Achard , P. Arnoux , M. Barberi‐Heyob , J.‐C. Andre , X. Allonas , F. Baros , R. Vanderesse , Curr. Med. Chem. 2012, 19, 5580; 22978328 10.2174/092986712803833128

[46] J. Chen , K. Stefflova , M. J. Niedre , B. C. Wilson , B. Chance , J. D. Glickson , G. Zheng , J. Am. Chem. Soc. 2004, 126, 11450. 15366886 10.1021/ja047392k

[47] M. Zhang , Z. Zhang , D. Blessington , H. Li , T. M. Busch , V. Madrak , J. Miles , B. Chance , J. D. Glickson , G. Zheng , Bioconjug. Chem. 2003, 14, 709. 12862422 10.1021/bc034038n

[48] G. Zheng , J. Chen , K. Stefflova , M. Jarvi , H. Li , B. C. Wilson , Proc. Natl. Acad. Sci. USA 2007, 104, 8989. 17502620 10.1073/pnas.0611142104 PMC1868591

[49] P.‐C. Lo , J. Chen , K. Stefflova , M. S. Warren , R. Navab , B. Bandarchi , S. Mullins , M. Tsao , J. D. Cheng , G. Zheng , J. Med. Chem. 2008, 52, 358. 10.1021/jm801052f PMC2773291 19093877

[50] a) R. Kalluri , M. Zeisberg , Nat. Rev. Cancer 2006, 6, 392; 16572188 10.1038/nrc1877

[51] E. Cló , J. W. Snyder , N. V. Voigt , P. R. Ogilby , K. V. Gothelf , J. Am. Chem. Soc. 2006, 128, 4200. 16568974 10.1021/ja058713a

[52] K. V. Gothelf , A. Thomsen , M. Nielsen , E. Cló , R. S. Brown , J. Am. Chem. Soc. 2004, 126, 1044. 14746471 10.1021/ja038333u

[53] H. Chen , J. Tian , W. He , Z. Guo , J. Am. Chem. Soc. 2015, 137, 1539. 25574812 10.1021/ja511420n

[54] a) Y. Cho , H. Kim , Y. Choi , Chem. Commun. 2013, 49, 1202; 10.1039/c2cc36297j 23283113

[55] F. Li , S. J. Park , D. Ling , W. Park , J. Y. Han , K. Na , K. Char , J. Mater. Chem. B 2013, 1, 1678. 10.1039/c3tb00506b 32260699

[56] B. Tian , C. Wang , S. Zhang , L. Feng , Z. Liu , ACS Nano 2011, 5, 7000. 21815655 10.1021/nn201560b

[57] J. Xu , F. Zeng , H. Wu , C. Yu , S. Wu , ACS Appl. Mater. Interfaces 2015, 7, 9287. 25876183 10.1021/acsami.5b02297

[58] L. Liu , L. Fu , T. Jing , Z. Ruan , L. Yan , ACS Appl. Mater. Interfaces 2016, 8, 8980. 27020730 10.1021/acsami.6b01320

[59] a) Y. Chen , P. Xu , Z. Shu , M. Wu , L. Wang , S. Zhang , Y. Zheng , H. Chen , J. Wang , Y. Li , Adv. Funct. Mater. 2014, 24, 4386;

[60] J. Zhao , L. Huang , X. Cui , S. Li , H. Wu , J. Mater. Chem. B 2015, 3, 9194. 10.1039/c5tb01857a 32263135

[61] a) Z. Yang , J. H. Lee , H. M. Jeon , J. H. Han , N. Park , Y. He , H. Lee , K. S. Hong , C. Kang , J. S. Kim , J. Am. Chem. Soc. 2013, 135, 11657; 23865715 10.1021/ja405372k

[62] J. Han , W. Park , S. J. Park , K. Na , ACS Appl. Mater. Interfaces 2016, 8, 7739. 26965036 10.1021/acsami.6b01664

[63] S. Erbas‐Cakmak , O. A. Bozdemir , Y. Cakmak , E. U. Akkaya , Chem. Sci. 2013, 4, 858.

[64] a) O. AltanáBozdemir , Chem. Sci. 2013, 4, 858;

[65] a) L. Zhang , A. M. Bluhm , K. J. Chen , N. E. Larkey , S. M. Burrows , Nanoscale 2017, 9, 1709; 28090611 10.1039/c6nr07814a

[66] a) J.‐c. Qin , Z.‐y. Yang , J. Photochem. Photobiol. A 2016, 324, 152;

[67] L. Huang , W. Yang , J. Zhao , J. Org. Chem. 2014, 79, 10240. 25279767 10.1021/jo5019014

[68] a) S. O. McDonnell , M. J. Hall , L. T. Allen , A. Byrne , W. M. Gallagher , D. F. O'shea , J. Am. Chem. Soc. 2005, 127, 16360; 16305199 10.1021/ja0553497

[69] a) O. Nevskyi , D. Sysoiev , A. Oppermann , T. Huhn , D. Wöll , Angew. Chem. Int. Ed. 2016, 55, 12698; 10.1002/anie.201606791 27619176

[70] L. Hou , X. Zhang , T. C. Pijper , W. R. Browne , B. L. Feringa , J. Am. Chem. Soc. 2014, 136, 910. 24392882 10.1021/ja4122473

[71] J. Karolczak , D. Kowalska , A. Lukaszewicz , A. Maciejewski , R. P. Steer , J. Phys. Chem. A 2004, 108, 4570.

[72] C. Fan , S. Pu , G. Liu , Dyes and Pigments 2015, 113, 61.

[73] Y. Liu , G. Liu , X. Xu , Y. Chen , X. Wu , H. Wu , Chem. Commun. 2016, 52, 7966. 10.1039/c6cc02996e 27251874

[74] J. Park , D. Feng , S. Yuan , H. C. Zhou , Angew. Chem. Int. Ed. 2015, 54, 430. 10.1002/anie.201408862 25476702

[75] J. Park , Q. Jiang , D. Feng , H. C. Zhou , Angew. Chem. Int. Ed. 2016, 128, 7304.

[76] a) S. S. Razi , R. Ali , R. C. Gupta , S. K. Dwivedi , G. Sharma , B. Koch , A. Misra , J. Photochem. Photobiol. A 2016, 324, 106;

[77] a) Y. M. Zhang , W. J. Qu , G. Y. Gao , B. B. Shi , G. Y. Wu , T. B. Wei , Q. Lin , H. Yao , New J. Chem. 2014, 38, 5075;

[78] a) Y. Miao , B. Zhao , Z. Gao , H. Shi , P. Tao , Y. Wu , K. Wang , H. Wang , B. Xu , F. Zhu , Org. Electron. 2017, 42, 1;

[79] a) L. Vachova , V. Novakova , K. Kopecky , M. Miletin , P. Zimcik , Dalton Trans. 2012, 41, 11651; 22832706 10.1039/c2dt31403g

[80] X. F. Zhang , X. Yang , J. Phys. Chem. B 2013, 117, 9050. 23837434 10.1021/jp405102m

[81] a) W. Pang , X. F. Zhang , J. Zhou , C. Yu , E. Hao , L. Jiao , Chem. Commun. 2012, 48, 5437; 10.1039/c2cc30915g 22531223

[82] K. Hirakawa , Y. Nishimura , T. Arai , S. Okazaki , J. Phys. Chem. B 2013, 117, 13490. 24144045 10.1021/jp4072444

[83] a) D. Margulies , C. E. Felder , G. Melman , A. Shanzer , J. Am. Chem. Soc. 2007, 129, 347; 17212414 10.1021/ja065317z

[84] S. Erbas‐Cakmak , E. U. Akkaya , Angew. Chem. Int. Ed. 2013, 52, 11364. 10.1002/anie.201306177 24030974

[85] X. S. Li , M. R. Ke , W. Huang , C. H. Ye , J. D. Huang , Chem‐Eur. J. 2015, 21, 3310. 25639348 10.1002/chem.201404514

[86] a) K. Ma , R. Xing , T. Jiao , G. Shen , C. Chen , J. Li , X. Yan , ACS Appl. Mater. Interfaces 2016, 8, 30759; 27778498 10.1021/acsami.6b10754

[87] K. Liu , R. Xing , Q. Zou , G. Ma , H. Mohwald , X. Yan , Angew. Chem. Int. Ed. 2016, 55, 3036. 10.1002/anie.201509810 26804551

[88] J. Tan , C. Sun , K. Xu , C. Wang , J. Guo , Small 2015, 11, 6338. 26514273 10.1002/smll.201502131

[89] D. Ling , W. Park , S. J. Park , Y. Lu , K. S. Kim , M. J. Hackett , B. H. Kim , H. Yim , Y. S. Jeon , K. Na , T. Hyeon , J. Am. Chem. Soc. 2014, 136, 5647. 24689550 10.1021/ja4108287

[90] a) N. J. Turro , F. C. Ming , J. Am. Chem. Soc. 1981, 103, 7218;

[91] J. Xu , S. Sun , Q. Li , Y. Yue , Y. Li , S. Shao , Analyst 2015, 140, 574. 25422882 10.1039/c4an01934b

[92] C. Robertson , D. H. Evans , H. Abrahamse , J. Photochem. Photobiol. B 2009, 96, 1. 19406659 10.1016/j.jphotobiol.2009.04.001

[93] S. Kolemen , T. Ozdemir , D. Lee , G. M. Kim , T. Karatas , J. Yoon , E. U. Akkaya , Angew. Chem. Int. Ed. 2016, 55, 3606. 10.1002/anie.201510064 26845734

[94] I. S. Turan , D. Yildiz , A. Turksoy , G. Gunaydin , E. U. Akkaya , Angew. Chem. Int. Ed. 2016, 55, 2875. 10.1002/anie.201511345 26799149

[95] K. Lou , J. F. Lovell , Chem. Commun. 2014, 50, 3231. 10.1039/c3cc49171d 24522513

